# Recent Advances in Pathogenesis, Diagnostic Imaging, and Treatment of Sjögren’s Syndrome

**DOI:** 10.3390/jcm13226688

**Published:** 2024-11-07

**Authors:** Yoshiro Horai, Toshimasa Shimizu, Hideki Nakamura, Atsushi Kawakami

**Affiliations:** 1 Department of Rheumatology, Sasebo City General Hospital, 9-3 Hirase-cho, Sasebo 857-8511, Japan; 2 Department of Immunology and Rheumatology, Division of Advanced Preventive Medical Sciences, Nagasaki University Graduate School of Biomedical Sciences, Nagasaki 852-8523, Japan; toshimasashimizu2000@yahoo.co.jp (T.S.); atsushik@nagasaki-u.ac.jp (A.K.); 3 Clinical Research Center, Nagasaki University Hospital, Nagasaki 852-8501, Japan; 4 Division of Hematology and Rheumatology, Department of Medicine, Nihon University School of Medicine, Tokyo 173-8610, Japan; nakamura.hideki@nihon-u.ac.jp

It is our pleasure to present the Special Issue “Diagnosis and Treatment of Sjögren’s Syndrome” to the readers of the *Journal of Clinical Medicine*. This Special Issue comprises three original articles and two reviews covering recent developments in the understanding of the pathophysiology and management of Sjögren’s syndrome (SS).

SS is a rheumatic disease exhibiting dry eye and dry mouth due to the lymphocytic infiltration of the exocrine glands as its two most common symptoms [1,2,3,4,5,6,7,8,9,10]. It is classified into primary SS (pSS) and secondary SS based on the presence or absence of rheumatic diseases other than SS [11]. SS is considered a multifactorial disease involving both genetic and environmental factors. Viral infections, such as Epstein–Barr virus infection, have been well studied among the presumed environmental factors contributing to the onset of SS [12,13]. Additionally, human T-cell leukemia virus type I is another widely known microorganism involved in the pathophysiology of SS. Toll-like receptors (TLRs) play important roles in innate immunity and host defense against viral infections [14,15]. Epigenetic processes involving noncoding RNAs (ncRNAs) have recently been investigated as pathological factors in SS [16,17,18,19,20,21,22]. Several kinds of long ncRNAs (lncRNAs), a type of noncoding RNA with ≥200 nucleotides, are associated with the control of viral infection, modulating interferon responses that could be induced by TLR signaling. Based on this accumulated knowledge and current progress, we wrote a comprehensive review on pSS, covering aspects of viral infections, TLRs, and lncRNAs, which could help in the exploration of molecular therapeutic options [23].

Diagnostic imaging techniques, such as parotid sialography and salivary scintigraphy, were adopted as diagnostic criteria in the 2002 American-European Consensus Group SS classification criteria [11]. However, these present disadvantages such as radiation exposure and difficulties in the procedure and were not, therefore, included in the 2016 American College of Rheumatology/European Alliance of Associations for Rheumatology (formerly European League Against Rheumatism) classification criteria for SS. Recent advancements in diagnostic imaging, such as ultrasound (US) and magnetic resonance imaging (MRI), despite not having been adopted as diagnostic items in the 2016 SS classification criteria [24], enable not only early detection, but also the grading of the damage caused by SS in major salivary glands (SGs) [25,26]. US is a non-invasive, radiation-free imaging technique, which has the potential to advance the diagnostic procedure of SS. Moreover, Doppler US score was shown to be effective for evaluating vascularization in major SGs in a study of Outcome Measures in Rheumatology US subgroup on SS [27]. MRI is thought to be useful for detecting abnormal findings in SGs [28]. Takagi et al. clarified the occurrence rates of ranulas and parotid cysts on MRI, and parotid calcifications on computed tomography (CT) in definite SS, possible SS, and non-SS, and found that the presence of a ranula would help diagnose SS at an early stage [29].

SS is not generally regarded as a life-threatening disease because its prominent manifestations, sicca symptoms, are mainly treated with symptomatic therapy [30,31]. However, SS can also involve extraglandular damage that may affect vital organs. Pulmonary involvement is one of the most prevalent extraglandular organ damage occurrences and is associated with higher mortality in patients with SS [32,33]; it is adopted as a scoring item in the European Alliance of Associations for Rheumatology (formerly European League Against Rheumatism) SS Disease Activity Index [34]. Therefore, early and appropriate diagnosis and treatment of pulmonary involvement are important in the management of SS. La Rocca et al. contributed a detailed and up-to-date review of the pulmonary involvement in pSS to this Special Issue. Diagnostic imaging is useful not only for detecting glandular damage, as described above, but also for detecting pulmonary involvement in SS. In a review, La Rocca et al. explained that lung US and high-resolution chest CT are high-sensitivity methods for detecting pulmonary involvement in pSS. Other than diagnostic imaging, biomarkers that could be related to pulmonary involvement in SS are also listed in the review: anti-Ro/SS-A and La/SS-B antibodies, C-reactive protein levels, hypergammaglobulinemia, and high focus scores in SG biopsy [35], which indicates that pulmonary involvement has a close relationship with systemic disease activity. The findings of the study contributed to the Special Issue by Roman et al. brought new insights into correlations between serum biomarkers and high-resolution chest CT findings on lung involvement in pSS, and a positive correlation was observed between interleukin-8 levels and Warick scores (a method employed to evaluate pulmonary involvement borrowed from a study on systemic sclerosis-associated interstitial lung disease) [36,37].

The physical burden of dry eye is not thought to affect life expectancy but is profound, causing restrictions on daily activities. Dry eye in SS also has a negative impact on work productivity [38]; therefore, the development of effective treatments for dry eye is significant for improving both the quality of life of patients with SS and economic effects. To date, symptomatic therapies such as tear drops are the mainstay treatment for dry eye in SS [30,31]. However, widely used artificial tear substitutes, which differ from natural tears in terms of deficiencies in proteins, lipids, and growth factors, and the addition of preservatives, do not always produce satisfactory effects in patients with SS. These problems can be overcome by blood-derived eye drops, such as autologous serum and platelet-rich plasma, as described in a comparative study of dry eye in pSS by Wróbel-Dudzińska et al. [39].

We hope that the findings in this Special Issue will be of special interest to readers, and that the future perspectives covered in the papers will be investigated in further research.
